# Reach and Cost-Effectiveness of the PrePex Device for Safe Male Circumcision in Uganda

**DOI:** 10.1371/journal.pone.0063134

**Published:** 2013-05-22

**Authors:** Kevin Duffy, Moses Galukande, Nick Wooding, Monica Dea, Alex Coutinho

**Affiliations:** 1 International Medical Group, Kampala, Uganda; 2 International Hospital Kampala and Surgery Department, Mulago Hospital, Kampala, Uganda; 3 International Health Sciences University, Kampala, Uganda; 4 Centers for Disease Control, Kampala, Uganda; 5 Infectious Diseases Institute, Makerere University, Kampala, Uganda; Yale School of Public Health, United States of America

## Abstract

**Introduction:**

Modelling, supported by the USAID Health Policy Initiative and UNAIDS, performed in 2011, indicated that Uganda would need to perform 4.2 million medical male circumcisions (MMCs) to reach 80% prevalence. Since 2010 Uganda has completed 380,000 circumcisions, and has set a national target of 1 million for 2013.

**Objective:**

To evaluate the relative reach and cost-effectiveness of PrePex compared to the current surgical SMC method and to determine the effect that this might have in helping to achieve the Uganda national SMC targets.

**Methods:**

A cross-sectional descriptive cost-analysis study conducted at International Hospital Kampala over ten weeks from August to October 2012. Data collected during the performance of 625 circumcisions using PrePex was compared to data previously collected from 10,000 circumcisions using a surgical circumcision method at the same site. Ethical approval was obtained.

**Results:**

The moderate adverse events (AE) ratio when using the PrePex device was 2% and no severe adverse events were encountered, which is comparable to the surgical method, thus the AE rate has no effect on the reach or cost-effectiveness of PrePex. The unit cost to perform one circumcision using PrePex is $30.55, 35% ($7.90) higher than the current surgical method, but the PrePex method improves operator efficiency by 60%, meaning that a team can perform 24 completed circumcisions compared to 15 by the surgical method. The cost-effectiveness of PrePex, comparing the cost of performing circumcisions to the future cost savings of potentially averted HIV infections, is just 2% less than the current surgical method, at a device cost price of $20.

**Conclusion:**

PrePex is a viable SMC tool for scale-up with unrivalled potential for superior reach, however national targets can only be met with effective demand creation and availability of trained human resource.

## Introduction

In 2007 the World Health Organisation (WHO) and UNAIDS reported that RCT studies had shown male circumcision (MC) could reduce the risk of HIV infection in men by about 60%. The report recommended medical male circumcision (MMC) as an integral part of comprehensive HIV prevention programmes [1], [2], [3], [4].

In 2011 Njeuhmeli and a team of experts created a model to demonstrate the costs and impacts of large scale national MC programmes across 13 countries in eastern and southern Africa. The proposed strategy was to first reach a MC prevalence of 80% during a catch-up period of 5 years to 2015 and to then maintain this level through to 2025. They estimated that this would require a total of 28.7 million circumcisions, cost about $2 billion and that doing so could help to prevent up 3.3 million new HIV infections, saving more than $16.5 billion in future costs of treatment [5].

The same modelling shows that Uganda will need to perform 4.2 million MCs to reach 80% prevalence by 2015 and an additional 2.1 million to maintain that prevalence through to 2025 [5], [6], [7]. During 2011/12 a number of Implementation Partners (IPs) conducted pilot programmes and started to plan for scale-up in 2012/13 (October – September). The Uganda Ministry of Health has set a national target of 1 million circumcisions to be performed in 2012/13. Reports issued during World AIDS Day 2012 indicated that since the start of its SMC Programme in 2010 Uganda has completed 380,000 circumcisions, of which 348,099 were performed in the 12 months up to September 30^th^ 2012. Reaching the 2012/13 target will require a three fold increase in output.

To date, circumcisions have been performed using the surgical methods prescribed by the WHO. Studies using non-surgical circumcision devices are underway. Such devices include PrePex, an elastic ring controlled radial compression device that causes necrosis of the foreskin in seven days or less. The PrePex device enables safe non-surgical male circumcision. Efficacy, Safety and Comparative studies have been completed in Rwanda and will soon be completed in Zimbabwe. No studies have yet been concluded in Uganda [8], [9], [10], although two are under way at IHK and Rakai.

PrePex is a non-surgical circumcision device, developed by Circ MedTech. A plastic ring is inserted inside the foreskin and a rubber ring is placed on the outer foreskin, on top of the inner ring, stopping the flow of blood to that part of the foreskin that is to be removed. After 7 days the foreskin becomes necrotic and can be removed simply by cutting with scissors; no anaesthesia or sutures are required. Placement and removal of PrePex can be performed in a non-sterile setting.

International Medical Group (IMG) gained approval to conduct a Safety Study to consider the use of the PrePex device by Physicians and Non Physician Clinicians (NPCs), acceptance of the method by patients and the relative significance of any resulting AEs when compared with a surgical method.

The aim of this related cost-analysis study was to provide average unit cost and cost-effectiveness information related to performing safe male circumcision using either a surgical method or PrePex and to determine whether use of PrePex might enhance the delivery of the national SMC targets.

### Study Site Context

In the period April 2011 to June 2012 IHK, a private IMG hospital, performed more than 10,000 adult SMCs, mostly using the sleeve resection method. A review of the data collected during the first 4 months (April – July 2011) showed that the average work rate was 14 SMCs per table or pair of operators (NPCs) per day. This started as 7 and rose to 23 over time as staff gained more experience. The time per procedure dropped from an initial average of 55 minutes to just 29 minutes. It was noted that an experienced pair could perform a SMC using this method in just 14 minutes. These fastest times should not be extrapolated to indicate expected outputs over much longer, regular ongoing periods. After 10,000 procedures IHK finds a regular output per pair per working day of 15–18 SMCs. The best pairs are able to perform up to 20 circumcisions in a day, but not all pairs are able to achieve this on a consistent basis. Thus IHK has set the target output by one pair, per working day, at 15 SMCs [11].

## Methods

### Study Design

This is a cross-sectional descriptive cost-analysis study.

The study first established the time taken to perform one circumcision and then the total number of circumcisions that can be performed by a pair of staff in a full working day.

Unit costs were established by compiling each of the ingredient costs incurred using a bottom-up approach and combining these with those overhead and shared costs that needed to be apportioned. This is consistent with the approach recommended in “Analysis of Hospital Costs: A Manual for Managers” [12] and is similar to that needed when using the DMPPT [13].

Cost-effectiveness was determined by comparing the unit cost of a circumcision with the estimated discounted savings of future care and treatment costs avoided from those HIV infections averted as a result of the circumcision. This is the approach first described in 2006 and followed by others since [14].

When reviewing the efficiency of the operational process and the potential impact of PrePex, the study took into account matters such as the availability of adequately credentialed staff, the time taken for new staff to become proficient in the method and the facility and equipment requirements. These address some of the questions raised by the WHO Technical Advisory Group on Innovations in Male Circumcision (TAG) about the potential usefulness of the PrePex innovation in easing greater acceleration of national programmes [15].

### Setting

This study was carried out at International Hospital Kampala (IHK), a 120 bed private hospital located in an urban setting, close to the central business district of Kampala. IHK has space and equipment, including 16 procedure tables, dedicated to its SMC Programme which started in April 2011. From that time to December 2012 the IHK team has performed more than 14,000 SMCs using the sleeve resection method. The PrePex placements and removals were performed in non-sterile rooms, which were dedicated to this purpose.

### Study population

All adult males, aged 18 years and above, presenting themselves voluntarily for SMC.

### Sample Size

Data from the previous 10,000 SMCs was compared to the prospectively collected data for 625 clients.

### Sampling

For PrePex timings a convenient consecutive sampling approach was taken.

### Sources of data

Primary financial and quantitative data were collected and collated during the 625 circumcisions using PrePex.

Secondary financial and quantitative data related to the 10,000 circumcisions performed at IHK were taken from its audited financial reports and monitoring reports.

Costs of consumables and reusable sets were confirmed by reference to the current Joint Medical Stores (JMS) (a private firm in Uganda dealing in medical supplies) price list.

Data informing the number of MCs necessary in Uganda for one HIV infection averted (HIA) and the lifetime cost of a HIV infection were taken from the 2011 DMPPT model [5], [13].

Data related to the numbers of health workers in Uganda are taken from the World Health Statistics, 2012 [16].

### Study variables

Human resource count and costs per cadre and role, quantities and costs of medical consumables, quantities and costs of reusable equipment, cost of the PrePex device, overheads and shared costs, operator outputs and study timings.

### Data collection

The time taken to perform a circumcision using the surgical method was derived from existing data files and confirmed in key informant interviews.

The times taken to perform circumcisions using PrePex were collected into a specifically designed form through observation and use of a stopwatch. Timing started when a client lay on the table and stopped when he got back off the table. The time between that client leaving and the next client moving on to the bed was also recorded. Timing continued when there were any unplanned interruptions, which were sometimes due to a client having an erection or needing to relax and compose himself after a painful removal.

Data related to staff roles and numbers, and the quantities of equipment and consumables required, were gathered during key informant interviews. Data files were reviewed together during these interviews to ensure these were complete and correct.

All other financial and quantitative data were collected through desk research and the analysis of source data files provided by others.

### Quality control

Timings for PrePex were collected on 4 days, over a 2 week period and measured a total of 75 placements and 75 removals. This number of timings was considered to be sufficient when it was noted that there was no resulting change in the average number per day, derived from the first 3 days of data, when timings from day 4 were included.

Financial data were sourced from and verified by reference to the 2011 IMG audited accounts and 2012 management accounts.

Costs of consumables and reusable equipment were sourced from and verified by reference to the current Joint Medical Stores price list.

Unit costs of consumables were derived from actual consumption reports rather than procurement reports.

The costs for sterilisation of sets at IHK were compared with published costs for the same process at another similar facility in Kampala [17].

### Data analysis

Microsoft Excel 2007 was used to collate, analyse and compute all numeric and financial data.

### Ethical issues

Ethical approval was obtained from Makerere School of Medicine Research and Ethics Committee and the Uganda National Council of Science and Technology.

## Results

### Procedure timings

In order to complete a SMC using PrePex the client needs to attend the centre twice, initially for device placement and then returning after 7 days for device removal. The operations model for PrePex assumes that equal numbers of placements and removals will be performed by a pair of staff during each session. During placements both work at the table together. Only one is required to perform removals, leaving the other available to perform screening of new clients before placement. Timings per procedure improved quickly as newly trained staff gained experience and these began to plateau on the fourth day after which the average time to complete a placement was just over seven minutes (7 min 12 s), which included the time taken between clients when there was a steady flow and other clients were waiting. The equivalent for removal was ten minutes (10 min 7 s). A pair had two hours and fifty five minutes (2:55:00) placement time and four hours and five minutes (4:05:00) removal time in a 7 hour working day and completed 24 circumcisions. The skin-to-skin procedure times of about three minutes were consistent with the data presented by Circ MedTech, the device manufacturer.

### The Cost Model

These costs were modelled assuming a fully utilised centre operating 16 tables, performing circumcisions 4 days per week for a total of 46 weeks per year.

The exchange rate used in the above table is 2,515.877, which was sourced from the Bank of Uganda, Interbank Foreign Exchange Market (IFEM) mid-rate for September 2012.

In table 1, data in the PrePex Method column are primary data gathered during this study. Those data in the Surgical Method column are secondary data analysed and compiled for the purposes of this study.

**Table 1:** Cost model at a high volume SMC urban site at IHK, Uganda PrePex study 2012.

Item	Surgical	PrePex
Circumcisions Performed	44,160	70,656
Operator Staff Costs	$350,042	$350,042
Support Staff Costs	$82,026	$59,092
Cost of Consumables	$404,064	$216,349
Cost of Reusable Sets	$25,952	$4,868
Cost of Sterilisation	$48,134	$19,206
Cost of Devices	$0	$1,413,120
Non Staff Costs	$36,022	$41,786
Overheads and shared Costs	$53,872	$53,872
Total Costs	$1,000,112	$2,158,334
Unit Cost	$22.65	$30.55

The unit cost of a circumcision using PrePex is $30.55, which is 35% higher than the equivalent unit cost using the current surgical method, $22.65, a difference in this model of $7.90 per circumcision. This centre could perform an additional 26,496 circumcisions in one year using PrePex, an increase in output of 60% compared to the existing surgical method.

Operator staff were paid per day rather than per number of circumcisions performed, hence these costs were the same for both methods. The model reflected lower support staff costs for PrePex; the numbers for each staff role are shown in table 2. When using PrePex there were less supplies and equipment needed at each table, so the Runner is one less. The pieces within the reusable set number 3 for PrePex compared to 16 for the surgical method, the workload in the Central Sterile Supply Department (CSSD) was one person less. When using PrePex sterile linen was not needed and clients did not change out of their street clothes into a surgical gown, which meant less laundry and so one staff member less. After placement and removal the clients were discharged without the need to wait for some observation time in recovery, hence another staff number saved.

**Table 2:** Comparative HR needs in the SMC programme, Uganda PrePex study 2012.

Item	Surgical	PrePex
Operators	32	32
Supervisor and AE Management	1	1
Trainer	1	1
Runner	2	1
CSSD	2	1
Maintenance	2	2
Laundry	2	1
Recovery	1	0
Discharge	1	1
Registration	1	1
Customer Care	1	1
Supply Management	1	1
Data Management	1	1
After care on site cover	1	1
Counselling and Testing	3	3
Hot Line	3	3
	23	19

The analysis determined that the cost of consumables required in performing each circumcision using the surgical method was $9.15. Using PrePex required less, and different, consumables, for example there were no sutures or anaesthesia and examination gloves were used rather than sterile surgical gloves. The cost for this method was $3.06 per circumcision. The model uses these unit costs and factors in the extra circumcisions performed when using PrePex.

The cost of a reusable set for the surgical method was $58.77 compared with $6.89 for PrePex. The latter will require more sets due to the 60% extra procedures completed and this is factored in the model above. The model assumed that either set will have a reuse life of 100 times. Sets were sterilised within the existing IHK CSSD making use of underutilised autoclave capacity. The model used a cost of $1.09 for the sterilisation of a surgical set and factors in the smaller number of pieces for the PrePex set but also the larger number of sets required. Sterilization capacity existed at the study site.

The cost of PrePex is the manufacturer's list price of $20.00, no discount was available during this study.

Non staff costs include for example the use of airtime to perform client call back and the printing of client materials such as consent forms and hotline information. In the model this cost is higher for PrePex because the number of clients is higher.

Overheads and Shared Costs are those that have been apportioned to the circumcision programme based on its percentage use of overall facility space, equipment and utilities; also for central services such as hospital administration, HR, Finance and IT.

The cost of demand creation was not been included in this model. The success of the circumcision programme will depend very much on ensuring that centres are fully utilised and there are growing concerns that the number of men attending for circumcision is beginning to decline. Further research and analysis is required to establish best practice models that will maximise demand and inform the resulting cost for this.

IHK has a department which procures and manages all medical supplies, for all of its operations. This department procures from a range of local wholesale suppliers including Joint Medical Stores and Medical Access Uganda Limited. Department staff manage a warehouse of stock and transport supplies, when needed, to IHK. The cost of these supplies to IHK includes the cost of procurement and also all of the costs related to this department e.g. staff, rent, utilities and transport. This was the process used by the Study for the supply of all equipment and consumables required. Data used in the model and listed in Table 1 include the apportioned cost of this internal supply chain management.

### Cost-effectiveness

The 2011 DMPPT modelling indicated that Uganda needs to perform nineteen circumcisions in order to avert one future HIV infection and that the discounted lifetime cost of treatment is $7,400[5], [13].

The cost-effectiveness of a circumcision method can be measured by comparing its unit cost against the averted future costs of treatment [14].

Using the above, the cost-effectiveness of each method is:

Surgical: costs $430 (19×$30.55) for each HIA, with future cost savings of $6,970 ($7,400–$430).

PrePex: costs $580 (19×$30.55) for each HIA, with future cost savings of $6,820 ($7,400–$580).

PrePex has a unit cost 35% higher than the current surgical method. When future cost savings are taken into account PrePex is just 2% less cost-effective ((6,970–6,820)/6,920).

### Adverse Events

Eleven moderate adverse events occurred among 10 participants 11/625, (1.8%); these were all easily reversible. Six had device displacement, and five had bleeding after the device was removed. In addition to these, 15 participants made unscheduled visits 15/625 (2.4%) to the centre, seeking further guidance and assurance. These rates are comparable to those experienced when using the current surgical method and were managed by the clinical team performing the procedures, so the researchers determined that the AE rate and the number of unscheduled visits have no comparative effect on either reach or cost-effectiveness. These aspects of the PrePex method are discussed further in a manuscript in preparation by Galukande et al [18].

## Discussion

This study set out to investigate the cost-effectiveness and reach of the new non-surgical device, PrePex. We found that the unit cost per procedure using PrePex was 35% higher but offered a 60% superior reach when compared to the sleeve-resection surgical method.

Male circumcision can be viewed as an innovation in the prevention of HIV infection. The use of the PrePex device to perform circumcision could be viewed as an innovation within that innovation. Reach is an important aspect when considering any public health innovation [19], that is, will the innovation enable more services to be provided to more clients, more easily. Also will it be more accessible and more acceptable to the client. In March 2012, the WHO TAG issued a report recommending a phased implementation of PrePex in Rwanda and noted approval for the procedure to be performed there by NPCs. The report suggested that this innovation may contribute to a greater and easier acceleration of national MC programmes if it enables services to be provided more quickly, possibly at lower cost and make it easier for implementers to use lower cadres of staff, who are often more available in larger numbers [15].

This research has shown that PrePex can improve the rate at which teams can perform circumcisions, by as much as 60%. The PrePex method has a 35% higher unit cost but when future savings are taken into account the difference in cost-effectiveness is just 2% less. This margin could easily be changed by a reduction in the cost of the device or a review for potential cost savings or efficiency improvements in the operating process. It is logical to hypothesize that the daily output of 24 circumcisions per pair of staff could be improved by reducing the waiting time between clients by perhaps adopting a process in which staff work on multiple tables, rather than having downtime in between clients, similar to the MOVE model [20].

The PrePex method is not suitable for all clients and when given a choice not every client will choose PrePex over surgery. Research is required to more fully understand the reasons for these choices and the ratio that might elect for one method over the other. Further consideration is needed to determine if choice should be offered, particularly in the non-hospital settings. Each client attending for circumcision by the PrePex method is first screened to determine suitability for this procedure. Some clients have tight foreskins (phimosis) which make it difficult to insert the inner plastic ring and so these are not suitable for the procedure and are instead referred for the surgical method. It is probable that WHO pre-qualification will only be for those aged 18 years and above. For the purposes of this discussion we will assume that 70% of circumcisions are performed using the PrePex method and the balance by surgery, due to screen failure, client choice and age.

Uganda has set a target of 1 million SMCs in the year 2012/13. This cost model assumes that a pair of staff can perform circumcisions 4 days a week, 46 weeks in a year, so a total of 184 days. Based on that, and deploying only the surgical method, Uganda will need 725 full-time equivalents (FTEs) to meet this target. These 725 FTEs are just the staff performing and assisting the circumcision procedures, others will be required for the support functions as noted earlier.

Assuming that 70% of the procedures are performed using PrePex this number reduces by 36% to 534; 317 FTEs to use PrePex and 217 to perform the surgical method.

The World Health Statistics 2012 reports that there are 3,361 physicians in Uganda [16]. Under current regulations SMC must be performed by a physician, who can be assisted by a non-physician clinician. If these regulations remain unchanged, and only the surgical method is available, Uganda will need to dedicate 362, 11%, of its physician workforce full-time to perform SMC. It is difficult to understand how this will be possible, especially given the current discussions in government concerning the shortage of physicians to meet all of the current service demands. Using a blended model in which 70% of circumcisions are performed using PrePex, reduces this requirement to 267, 8%, which is still a substantial proportion of the physician workforce.

One alternative is to follow the lead set by Kenya and change the local regulations to allow specifically trained NPCs to perform these procedures [7]. There may be concerns about task shifting a surgical procedure to NPCs; these could be related to issues of patient safety and quality of care. The PrePex method does not require the injection of local anaesthesia, the cutting of live skin or the suturing of wounds; it is a non-surgical method that can be performed in a non-sterile setting. For these reasons it should be easier to consider task shifting SMC using PrePex to NPCs rather than the same consideration for the surgical method. The trial in Rwanda has shown that PrePex can be used safely and effectively by NPCs [10].

Changing the national regulations to allow specifically trained NPCs to perform SMC using PrePex would help to tackle the human resource issue. It is important to note that the regulations need to be explicitly changed in a manner which will enable organisations to procure professional indemnity insurance covering NPCs for such procedures.

There are 37,625 non-physician clinicians in Uganda^16^. If these were able to be deployed for PrePex then Uganda would only need to deploy 109 physicians and 426 NPCs, 3% and 1% of the respective total workforce numbers. Using NPCs could also present an opportunity to lower the unit cost of a circumcision, since these cadres are on lower pay scales.

It is likely that some service providers will want, or need, to blend SMC into the normal business as usual, rather than fully dedicating staff, facility space and equipment. This may be easier to do with PrePex than surgical. The latter needs a sterile setting and takes about 30 minutes per procedure. PrePex can be placed or removed in less than 10 minutes and needs nothing different than that which is normally available in a consultation room. The device method could therefore be more easily blended into a clinician’s normal routine, as it takes no more time and requires nothing especially different from many other primary care consultations.

PrePex does not require a sterile setting which will make it easier for an IP to set aside appropriate space for performing SMCs. There is less cost and effort involved in preparing such space, indeed nothing really needs to be done. Procedure tables are simple to acquire or construct and can be deployed in any available space. Staff do not need to be dressed in freshly laundered scrubs, nor do they need surgical masks or caps. Examination gloves are sufficient and are less expensive than sterile, surgical gloves. Clients do not need to change out of their clothes into surgical gowns; they simply drop their trousers and pants and get on the table. The reusable equipment set for PrePex has just three pieces, which means less to transport and a reduction in the required number of autoclave cycles. All of this makes the PrePex method very much easier to implement, especially in lower level health centres or in a non-clinical setting such as a school classroom or village hall. PrePex will be easier to deploy in a mobile or rural setting, which means that SMC services can be taken closer to the client, increasing accessibility. These are clear advantages in helping to reach the national targets.

There are a number of steps that can be taken when deciding if this innovation should be included into the guidelines and protocols for the safe male circumcision programme. Patient safety is paramount; a manuscript in preparation by Galukande et al [18] confirms that the AE rate for PrePex is comparable to that for the current surgical method. That manuscript also indicates that patients and operators find use of the device an acceptable method for MC and that staff are easily trained to use PrePex. This study has shown that deployment of the PrePex method will be easier operationally, and programmatically, than the sole use of a surgical method. So the PrePex method is safe, it is acceptable to patients and staff, it is easier to deploy at scale in multiple locations and settings, it enables a team to perform more circumcisions each day, but it has a higher unit cost. If the unit cost is paramount then negotiations will need to result in a device cost of $12 rather than the current $20 to bring the PrePex method into line with the current surgical method. Programme planners may however be willing to accept some extra unit cost in return for the opportunity to deploy more NPCs, simpler programme scale-up and the more rapid achievement of the required MC targets.

### Limitations of the study

This was done in a fixed location, high SMC volume urban site and so extrapolation of findings to a low volume rural site or mobile SMC model needs to be done with caution. The unit costs and thus the cost-effectiveness is very sensitive to resource utilisation rates. Staff costs are only semi-variable and if each role is not fully utilised the unit cost will increase. The costs are based on the current local prices which are subject to future inflation and exchange rate variations.

The success of the circumcision programme will depend very much on ensuring that centres are fully utilised and there are growing concerns that the number of men attending for circumcision is beginning to decline. This study did not evaluate the unit cost of demand creation, though this may not differ by SMC procedure method.

This study was performed from a service provider perspective and does not take into account the client time required or the costs incurred by the client receiving the service.

### Recommendations

This research has shown that the innovative PrePex device method will enable more services to be provided, more easily, potentially using lower cadres of staff without impacting on patient safety or quality of care. The innovation should make it more straightforward for the government and regulatory bodies to consider specific approval for the task shifting of this procedure, which will accelerate progress towards meeting the national targets. Whilst PrePex has a higher unit cost, its cost-effectiveness is almost the same as the surgical method. Deployment of PrePex may make it easier to reach scale in the rural setting, away from clinical facilities, increasing client accessibility.

The Ministry of Health and other regulatory bodies should move quickly to approve PrePex for use in Uganda’s SMC Programme alongside the surgical method as soon as the World Health Organisation has given its pre-qualification for widespread use.

### Conclusions

PrePex, a non-surgical SMC device, is overall cost-effective. Its benefit of superior reach may enhance actualization of the SMC targets in the 14 sub-Saharan countries subject to effective demand creation. Further field studies in rural and mobile contexts are needed urgently.
